# Can medical comorbidities modify the clinical evolution of mild cognitive impairment and Alzheimer’s dementia?

**DOI:** 10.1002/alz.086381

**Published:** 2025-01-09

**Authors:** Catherine Q N Nguyen, Liwei Ma, Yi Ling Clare Low, Edwin C.K. Tan, Christopher J Fowler, Colin L. Masters, Liang Jin, Yijun Pan, AIBL Research Group

**Affiliations:** ^1^ Florey Institute of Neuroscience and Mental Health, Parkville, VIC Australia; ^2^ University of Melbourne, Parkville, VIC Australia; ^3^ The University of Sydney, Sydney, NSW Australia; ^4^ Florey Institute, The University of Melbourne, Melbourne, VIC Australia; ^5^ Florey Institute of Neuroscience and Mental Health, University of Melbourne, Parkville, VIC Australia; ^6^ Tohoku University, Sendai Japan; ^7^ Monash University, Parkville, VIC Australia

## Abstract

**Background:**

Despite the health and societal burden that Alzheimer’s disease (AD) has on the elderly population, the underlying cause is not fully understood. Researchers are investigating possible mechanisms, and current studies have suggested that a number of comorbidities increase/decrease the likelihood of AD onset. The aim of the current study was to explore the associations between various comorbidities and AD in older Australians from an epidemiological perspective.

**Method:**

This study used secondary participant data from the Australian Imaging, Biomarker & Lifestyle (AIBL) study (n = 2436) to perform a quantitative analysis. Univariable logistic regression was used to quantify the relationship between comorbidities and mild cognitive impairment (MCI)/AD, represented as odds ratios (ORs). The top 20 comorbidities were investigated as the exposure and based on the participants’ medical histories. MCI and AD were the outcomes of interest (with cognitive unimpairment as the reference) and were based on participants’ cognitive health diagnoses. Selected comorbidity associations with MCI/AD were also analysed via multivariable logistic regression, by stratifying and adjusting for sex, age, education, smoking, drinking and *APOE* ε4 allele carrier status.

**Result:**

From the univariable logistic regression, the comorbidities with the strongest odds of developing AD in the AIBL cohort were anxiety, depression, neurological disorders, falls and stroke. Comorbidities with the most reduced odds of developing AD were kidney disease, liver disease and visual defects. These associations remained significant after multivariable logistic regression. When stratified for each covariate, several comorbidity associations observed effect modification from sex and *APOE* ε4 allele carrier status. When adjusted for all covariates, most of the selected comorbidity associations observed significant confounding. There were moderate similarities between the MCI and AD analyses.

**Conclusion:**

This study presents evidence that certain comorbidities appear to modify the clinical evolution of MCI/AD. Further research is needed to identify the mechanisms behind these associations and the potential modifying effect on MCI/AD.

